# A comparison of mantle versus involved-field radiotherapy for Hodgkin's lymphoma: reduction in normal tissue dose and second cancer risk

**DOI:** 10.1186/1748-717X-2-13

**Published:** 2007-03-15

**Authors:** Eng-Siew Koh, Tu Huan Tran, Mostafa Heydarian, Rainer K Sachs, Richard W Tsang, David J Brenner, Melania Pintilie, Tony Xu, June Chung, Narinder Paul, David C Hodgson

**Affiliations:** 1 University of Toronto, Department of Radiation Oncology, Princess Margaret Hospital, Toronto, Ontario, Canada; 2 University of Toronto, Department of Radiation Physics, Princess Margaret Hospital, Toronto, Ontario, Canada; 3 Department of Mathematics, University of California, Berkeley, California, USA; 4 Center for Radiological Research, Columbia University Medical Center, New York, New York, USA; 5 Department of Clinical Study Coordination and Biostatistics, Princess Margaret Hospital, Toronto, Ontario, Canada; 6 University of Toronto, Department of Medical Imaging, Princess Margaret Hospital, Toronto, Ontario, Canada

## Abstract

**Background:**

Hodgkin's lymphoma (HL) survivors who undergo radiotherapy experience increased risks of second cancers (SC) and cardiac sequelae. To reduce such risks, extended-field radiotherapy (RT) for HL has largely been replaced by involved field radiotherapy (IFRT). While it has generally been assumed that IFRT will reduce SC risks, there are few data that quantify the reduction in dose to normal tissues associated with modern RT practice for patients with mediastinal HL, and no estimates of the expected reduction in SC risk.

**Methods:**

Organ-specific dose-volume histograms (DVH) were generated for 41 patients receiving 35 Gy mantle RT, 35 Gy IFRT, or 20 Gy IFRT, and integrated organ mean doses were compared for the three protocols. Organ-specific SC risk estimates were estimated using a dosimetric risk-modeling approach, analyzing DVH data with quantitative, mechanistic models of radiation-induced cancer.

**Results:**

Dose reductions resulted in corresponding reductions in predicted excess relative risks (ERR) for SC induction. Moving from 35 Gy mantle RT to 35 Gy IFRT reduces predicted ERR for female breast and lung cancer by approximately 65%, and for male lung cancer by approximately 35%; moving from 35 Gy IFRT to 20 Gy IFRT reduces predicted ERRs approximately 40% more. The median reduction in integral dose to the whole heart with the transition to 35 Gy IFRT was 35%, with a smaller (2%) reduction in dose to proximal coronary arteries. There was no significant reduction in thyroid dose.

**Conclusion:**

The significant decreases estimated for radiation-induced SC risks associated with modern IFRT provide strong support for the use of IFRT to reduce the late effects of treatment. The approach employed here can provide new insight into the risks associated with contemporary IFRT for HL, and may facilitate the counseling of patients regarding the risks associated with this treatment.

## Background

It has long been established that Hodgkin's lymphoma (HL) survivors experience increased risks of secondary cancer (SC), in particular breast and lung cancer, and cardiac disease attributable in part to radiotherapy (RT) [1-6]. Most published estimates of SC risks after RT in HL survivors [5-8] are based on results from patients treated with extended-field RT, (that is, mantle, extended mantle or subtotal nodal RT fields that included both grossly enlarged lymph nodes as well as surrounding lymph nodes), which was widely used prior to the mid 1990s [9]. Since that time, in large part to reduce the risks of SC and cardiac toxicity, extended field radiotherapy for HL has generally been superceded by involved field radiation therapy (IFRT) delivered following chemotherapy [10]. Furthermore, reduced-dose IFRT (20 Gy) appears to produce comparable early disease control for selected favorable and intermediate risk patients, suggesting that this may become the standard adjuvant RT dose [11,12].

Since the advent of IFRT is relatively recent, there are few data to support or refute the assumption that reduced RT volumes will lead to a reduction in SC. A meta-analysis of 10 randomized trials found a significant reduction in the risk of breast cancer following IFRT compared to EFRT, but no significant reduction in the overall risk of all forms of SC combined [13]. Among 8 trials primarily involving early stage patients, there was a non-significant increase in SC rate among treatments that included EFRT (Odds Ratio, OR = 1.20, 95%CI = 0.88–1.62) [13]. Similarly, no difference in SC rate was found among 603 patients treated in British National Lymphoma Investigation (BNLI) Study [14].

A major limitation of standard observational studies of SC is the long latency required to observe the outcome and the resulting difficulty predicting the potential benefit associated with recent or potential future changes in practice (e.g. dose reduction to 20 Gy). An alternative, complimentary approach to these epidemiologic estimates of SC risk involves biologically-based modeling of SC risk. Until recently however, it was not practical to estimate SC risks after HL radiotherapy, because there was considerable uncertainty about the appropriate dose-responses for radiation-induced cancer at high radiation doses [15]. Older models of radiation carcinogenicity suggested that with increasing radiation doses above approximately 5 Gy, cellular killing offsets the induction of pre-malignancy, and the risk of developing radiation-induced SC declines [16,17]. However, these models are not compatible with the epidemiologic evidence among HL survivors, for whom the risk of SC continues to increase with increasing radiation doses above 30 Gy [5,7,8,18]. A recently developed mechanistically-based model of radiation carcinogenicity [19] provides estimates of second lung and breast cancer risk at high radiation doses (≥ 20 Gy) more compatible with epidemiological evidence [5,7,8].

The aims of this study were to quantify the reduction of radiation dose to normal tissues associated with the transition from 35 Gy mantle RT to 35 Gy IFRT to 20 Gy IFRT for patients with mediastinal HL, and to integrate this data in a radiobiological model to estimate the associated reductions in risk of radiotherapy-induced breast and lung cancer.

## Methods

Dose distributions were estimated for forty-one consecutive retrospectively identified patients with Stage I-III HL, who received mediastinal RT from January 2004 to July 2005 at the Princess Margaret Hospital, Canada. Pre-pubertal patients, those presenting with infradiaphragmatic disease only, and those receiving palliative RT, were excluded. Patient details are summarized in Table 1. All patients received chemotherapy prior to RT, most commonly ABVD (doxorubicin, bleomycin, vinblastine, dacarbazine). Approval from the research ethics board was obtained for this study.

**Table 1:** Description of baseline patient characteristics

Characteristics	Number
Gender	
Females	25 (61%)
Males	16 (39%)
Median age (range)	27 (14–58 years)
Smokers (current/ex-smoker)	14 (34%)
Pathology	
Nodular Sclerosing	39 (95%)
Nodular Lymphocyte Predominant	1 (2%)
Mixed Cellularity	1 (2%)
Stage I	4 (10%)
II	34 (85%)
III	2 (4%)
N/A (Relapse)	1 (2%)
Bulky disease *	28 (72%)
Chemotherapy Regimen	
ABVD	38 (93%)
Other	3 (7%)
Median cycles (range)	4 (3–8)
35 Gy IFRT plan – Treatment Indication	
Adjuvant	37
Post Transplant	3
Adjuvant Post-Relapse	1

* Bulky disease was defined as ≥ 5 cm on CT scan, and/or a thoracic ratio of maximum transverse mass diameter ≥ 33% of the internal transverse thoracic diameter measured at the T5/6 intervertebral disc level on chest radiography.

### Radiotherapy technique

**Figure 1:**
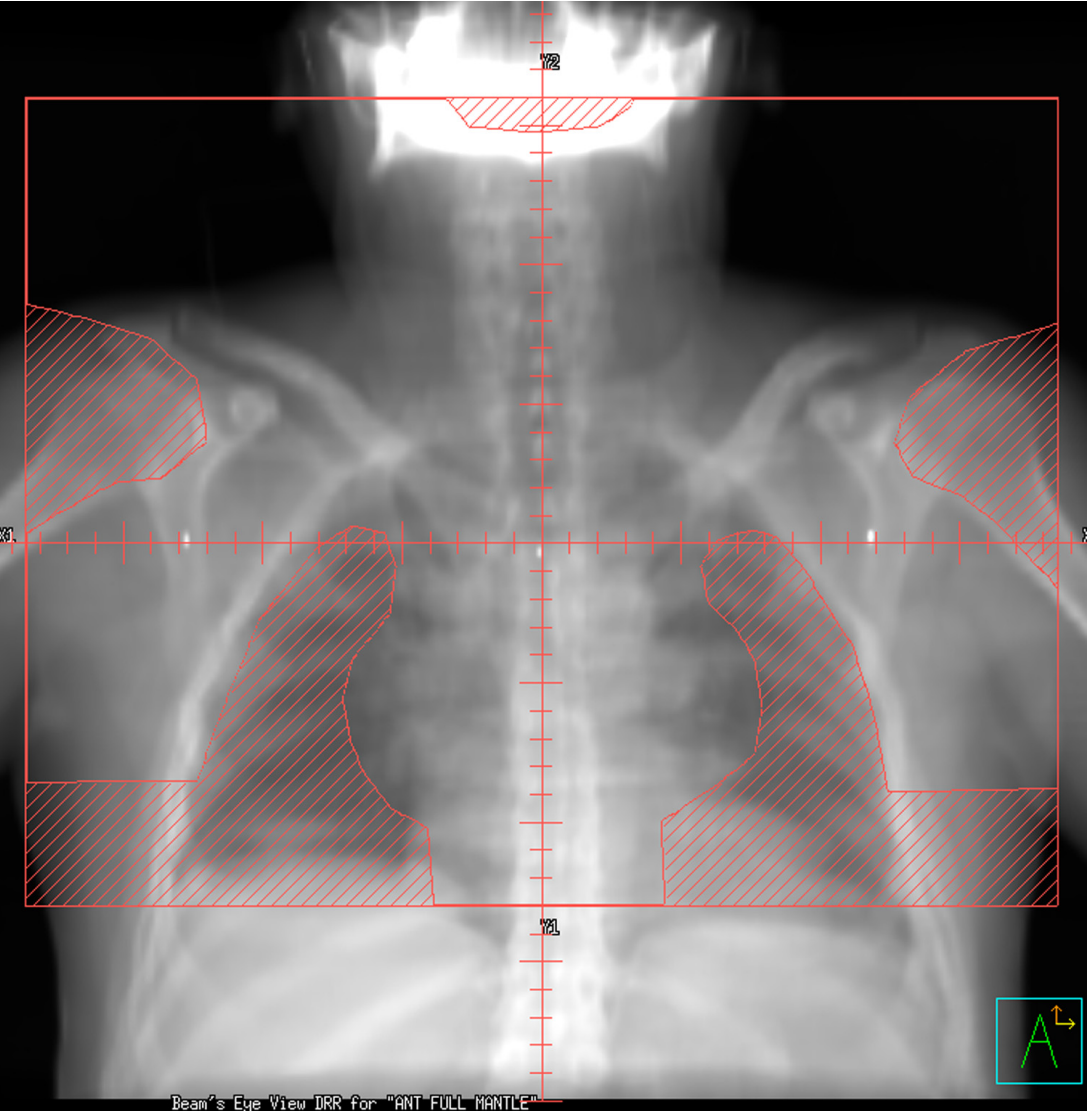
Digitally reconstructed radiographs demonstrating: mantle RT field (anterior beam shown).

**Figure 2:**
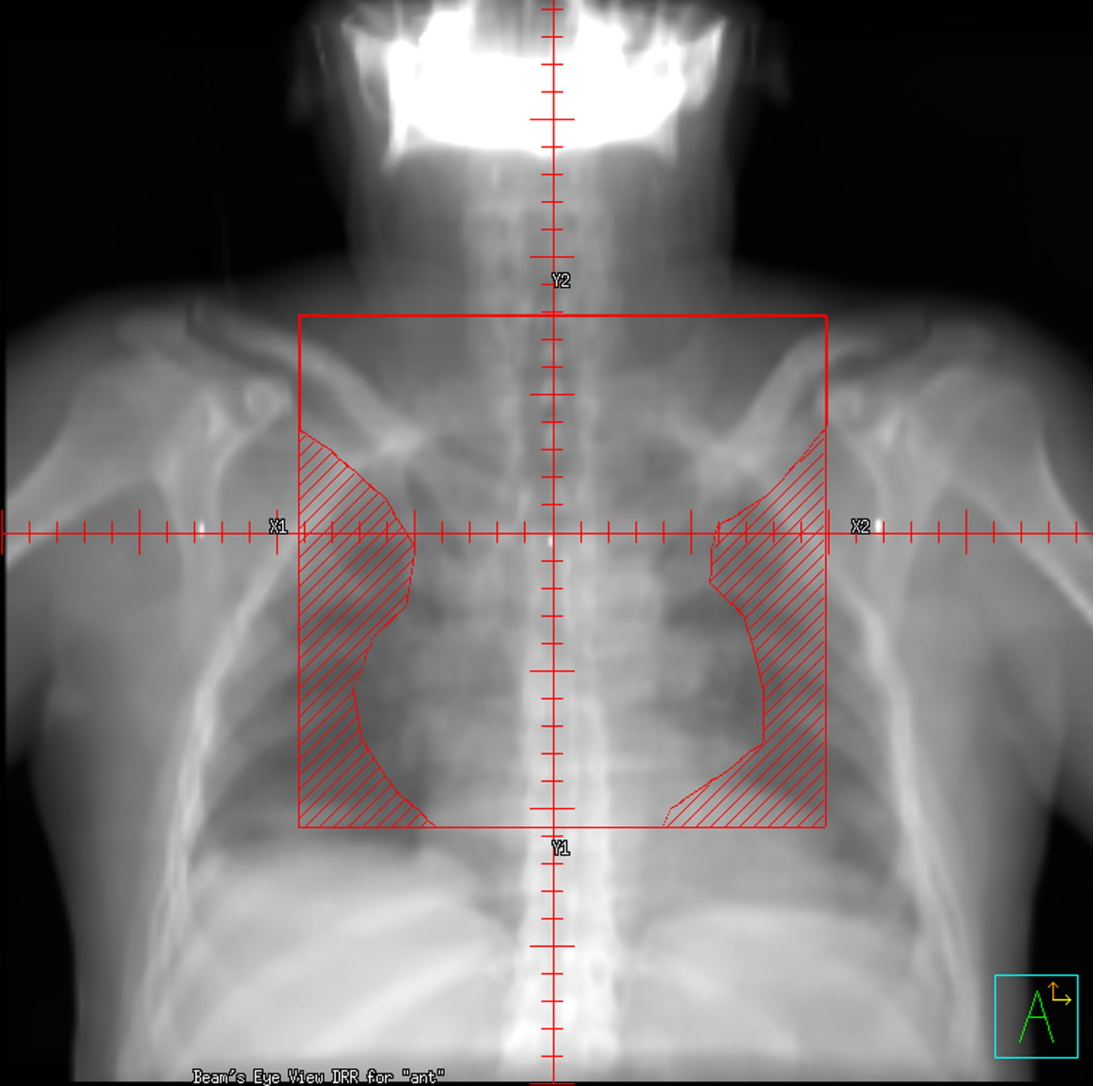
Digitally reconstructed radiographs demonstrating: mediastinal involved field RT (IFRT).

Patients were planned in the supine position, with neck extended, typically with arms akimbo and the upper torso immobilized in a Bodyfix^® ^device. For each patient, three treatment plans were constructed using the patient's planning CT data set: 35 Gy in 20 fractions mantle RT (historic treatment), 35 Gy in 20 daily fractions IFRT (current treatment), and 20 Gy in 10 daily fractions IFRT (potential future practice). Figures 1 and 2 show digitally reconstructed radiographs demonstrating typical RT field borders for 35 Gy mantle RT and IFRT, respectively.

For IFRT planning, clinical target volumes (CTV), planning target volumes (PTV), field borders and shielding were the same as those used for the actual IFRT delivered. The CTV typically consisted of the nodal regions involved with HL at the time of diagnosis, accounting for reduction in mediastinal width due to chemotherapy. Adjacent nodal regions were included in accordance with guidelines by Yahalom [20], with field borders as follows: upper border: C5-6 interspace (or at superior edge of larynx if supraclavicular nodes were involved); lower border: the lower of 50 mm inferior to the carina or 20 mm below the pre-chemotherapy inferior extent of disease; laterally: the post-chemotherapy volume with a 10–15 mm margin from CTV to shielding edge. Axillary RT was given only to axillary nodal groups that were involved at the time of diagnosis. The treatment volumes were identical for the 35 Gy and 20 Gy IFRT plans.

Mantle fields were designed according to accepted anatomic landmarks [20], extending from the mastoid process superiorly to the diaphragmatic insertion inferiorly, encompassing the bilateral axillae and extending laterally just beyond the humeral heads. Lung shields were placed 10–15 mm from the mediastinal contour and laterally followed the inner rib margins. Humeral head shielding throughout the RT course, as well as anterior laryngeal and posterior spinal cord shielding introduced at 24.5 Gy was planned. The cardiac dose was limited to = 30 Gy, below a transition zone located at 50 mm inferior to the carina.

For all treatment scenarios, the radiation field arrangement utilized opposed anterior and posterior beams, ensuring coverage of the CTV within ± 5% of the prescription dose, with point maximum doses within the treated volume no more than 110% of the prescription isodose accepted. All treatment plans were generated using the Pinnacle^® ^planning system, version 6.2b (ADAC Laboratories, Milpitas, CA).

### Calculating radiation dose to normal tissues

Contouring of the thyroid gland, bilateral female breasts, bilateral lungs, the whole heart, and the proximal coronary arteries (PCA) was performed under the supervision of a diagnostic radiologist (NP), and utilizing the cross-sectional anatomy illustrated in the Visible Human Project^®^datasets [21]. Given these organ contours, organ-specific differential dose volume histograms (DVH) were calculated using the Pinnacle treatment planning system. Integral organ doses were calculated by summing the DVH distributions, and mean doses as the ratio of integral dose to organ volume.

The percentage reductions in integral dose and mean dose to different organs associated with the transition from 35 Gy mantle RT to 35 Gy IFRT or 20 Gy IFRT was calculated for each patient. Differences in mean or integral organ doses, between the three protocols, were assessed using the Wilcoxon signed rank test. To quantify the reduction in the volume of breast and lung tissue exposed to low-dose radiation, we utilized dose-volume thresholds that have been previously associated with increased risks of secondary malignancy in HL patients: bilateral breast V_4 _(the volume of tissue receiving ≥ 4Gy) [5,8,22,23] and bilateral lung V_5 _[22]. V_30 _for the whole heart was also calculated [23].

### Second cancer risk modeling

Given a dose-volume histogram (DVH) for a normal tissue organ, and assuming each part of the organ in question is independent in terms of tumor initiation, the excess relative risk (ERR = Relative Risk (RR) -1) for organ-specific radiation-induced cancer induction can be estimated, provided that the dose-cancer-risk relation is known over the relevant dose range for that organ.

Dose-cancer-risk relationship at low and high radiation doses were obtained for breast and for lung, using a cell initiation/inactivation/proliferation model [19], which had previously been validated using recent radiation-induced second-cancer data in Hodgkin's disease patients treated with extended field RT [19]. This quantitative, mechanistic model of radiation-induced cancer risks is an extension of the standard initiation/inactivation cancer risk model [17]. Specifically the standard model predicts essentially zero radiation-related cancer risk at high doses, i.e. comparable to the prescribed tumor dose, due to radiation inactivation (killing) of radiation-initiated, pre-malignant cells. The more recent cancer-risk model [19], takes into account post-inactivation cellular repopulation by proliferation that occurs both during and after fractionated radiotherapy [24]. In terms of carcinogenesis, repopulation largely cancels out the effects of cellular inactivation, primarily because some of the proliferating cells carry and pass on pre-malignant damage produced earlier in the treatment. This extended model thus predicts substantial second-cancer risks even at doses as high as the prescribed tumor dose, consistent with the recent epidemiological data [5,7,8].

This cell initiation/inactivation/proliferation model [19] provides a practical approach to predicting organ-specific high-dose cancer risks based on a) cancer risk data from Atomic bomb survivors (who were exposed to lower doses), b) the demographic variables (age, time since exposure, gender, ethnicity) of the population/individual of interest, and c) two organ-specific parameters describing radiation-induced cellular repopulation, which have previously been estimated both for breast and lung [19]. First, ERRs are directly estimated for single radiation exposures at moderate doses, based on cancer incidence data among Atomic bomb survivors [25]. Second, a well established methodology described by Land and colleagues [26] (and almost identically in the recent BEIR-VII report [27] is used to adjust the dose-dependent ERRs from the Atomic bomb survivors to apply to the demographics (age, time since exposure, gender, ethnicity) of the individual(s) under study. These two steps were implemented through publicly available on-line software (Interactive RadioEpidemiological Program, IREP version 5.3) [28]. Finally, these moderate-dose ERR estimates for single exposures were adjusted to fractionated high-dose radiation exposure, using the initiation/inactivation/proliferation model [19] outlined above. The key parameter here, which has already been estimated for breast and lung [19], describes the ratio, *r*, of the per-cell proliferation rate for pre-malignant cells to the per-cell proliferation rate of normal cells. The values used in the present paper, slightly modified from those used earlier [19], are *r *= 1 for lung, and *r *= 0.825 for breast (values used earlier were *r *= 0.96 for lung, and *r *= 0.76 for breast, the current values give slightly better agreement with the earlier extended-field epidemiological data) [5,7]. For details regarding the modeling, including key assumptions, and mathematical estimation of ERR see Additional file 1.

Given the organ-specific ERR estimates for any given dose and fractionation scheme, the DVH data described above was used to estimate ERRs for radiotherapy-induced breast and lung cancer. In this "dosimetric + risk-modeling" method, each incremental small volume in the DVH, ΔV_j _(j = 1,200), is associated with a total dose D_j _= jΔD. Given the associated ERR (D_j_), estimated as described above, the overall predicted ERR is the volume-average of these local ERRs, i.e. ERR = (1/V)∑_j _ERR (D_j_) ΔV_j_, where V is organ volume. The modeling assumed that RT was given using fractions of prescribed daily dose = 1.75 Gy-2 Gy, with no treatment on weekends. ERR estimates would not vary significantly with changes in daily fraction size within a clinically realistic range.

In order to compare with results from the earlier extended-field radiotherapy, which is largely for prescription doses above 30 Gy [5,7,8], three representative patients were selected for analysis. These patients respectively had values for the mean female breast dose, mean female lung dose, and mean male lung dose that were closest to the median values of the whole group when treated with 35Gy mantle field RT (i.e. their radiation exposure with traditional RT fields and dose was the most representative). For each of these representative patients, ERR estimates were made for each of the three RT scenarios (35 Gy mantle field, 35 Gy IFRT, and 20 Gy IFRT).

## Results

### Radiation dose reduction

The median values among the 41 treated patients of the mean organ doses for the three treatment plans are shown in Table 2. Compared to 35 Gy mantle RT, the median mean organ doses from 35 Gy IFRT were significantly reduced (p < 0.001) for all studied organs except thyroid. Compared to 35 Gy mantle RT, 35 Gy IFRT reduced the median value of the mean dose to the female breast by 64%, the lung by 24%, the whole heart by 29%, and the proximal coronary arteries by 2%. The small but statistically significant reduction in mean dose to the proximal coronary arteries was largely attributable to 5 cases in which the CTV was located in the superior mediastinum, allowing the IFRT plans to reduce the mean dose to the PCA. The reductions in breast V_4_, lung V_5 _and cardiac V_30 _were 68%, 37% and 29% respectively.

**Table 2:** Mean radiation dose to normal tissues

	Thyroid (Gy)	Breast (Gy)	Lung (Gy)	Heart (Gy)	PCA* (Gy)
35 Gy Mantle (q1-q3)	34.4 (34.1–34.8)	9.0 (7.7–11.5)	14.7 (14.1–15.7)	24.2 (22.6–26.3)	34.7 (34.1–35.2)
35 Gy IFRT (q1-q3)	34.6 ^† ^(33.5–35.3)	3.2 (1.8–4.4)	11.2 (9.7–12.9)	17.2 (8.7–22.0)	33.9 (29.4–34.9)
20 Gy IFRT (q1-q3)	19.7 (19.2–20.2)	1.8 (1.0–2.6)	6.4 (5.5–7.3)	9.9 (5.0–13.2)	19.6 (17.2–20.0)

all figures quoted are median values, with first and third quartiles (q1-q3)

* PCA = proximal coronary arteries

† Compared to 35 Gy mantle RT, mean doses were significantly reduced (p < 0.001) for all organs with 35 Gy IFRT and 20 Gy IFRT, except for the mean dose to thyroid, which was not significantly reduced with 35 Gy IFRT.

**Figure 3:**
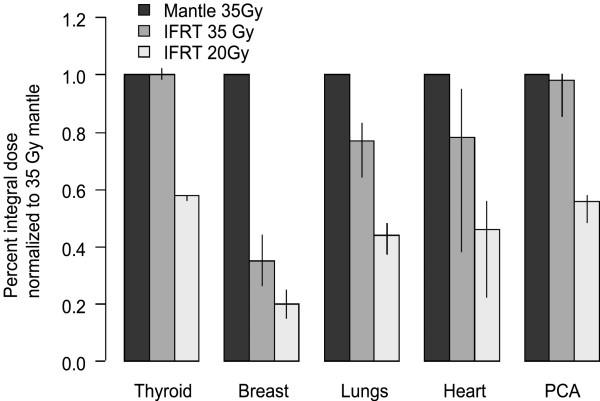
Proportional reduction in integral dose to normal tissues

As expected, reducing the IFRT prescription dose from 35 Gy to 20 Gy reduces all the mean organ doses by the same proportion, approximately 43%. Thus, compared with 35 Gy mantle, 20 Gy IFRT reduces the median value of the mean dose to the female breast by 80%, the lung by 56%, the whole heart by 59%, the proximal coronary arteries by 44%, and the thyroid by 43%. Reducing the prescribed IFRT dose from 35 Gy to 20 Gy produced a greater decrease in the mean dose to the PCA and the thyroid, than the change from mantle RT to IFRT. Breast V_4 _and lung V_5 _were reduced by 72% and 45% respectively. Figure 3 demonstrates the proportional reduction in integral dose to normal tissues for these three treatment scenarios.

### Second cancer risk reduction

Following RT for HL, breast and lung cancer account for the greatest burden of excess risk [1]. The estimated ERRs for radiation-induced breast cancer and lung cancer in never-smokers are shown in Table 3. The estimated age-specific ERRs at a time 20 years is shown after RT, but the relative reduction in ERRs (e.g. 35 Gy mantle vs. 35 Gy IFRT) would be unchanged for any other time post RT. Younger patients were predicted to have higher ERRs for SC than older patients, but similar proportional reductions in the ERR.

**Table 3:** Estimated excess relative risk (ERR*) of secondary breast and lung cancer 20 years after radiation exposure

	*Female Breast*		*Female Lung*		*Male Lung*	
	Age at RT (yrs)		Age at RT (yrs)		Age at RT (yrs)	
	20	30	20	30	20	30

35 Gy mantle RT (95% CI †)	4.6 (2.5–13.3)	2.1 (1.07–6.1)	18.4 (7.0–56.3)	7.6 (3.0–21.8)	12.6 (5.3–26.4)	5.2 (2.3–10.1)
35 Gy IFRT (95% CI)	1.7 (0.90–4.7)	0.74 (0.38–2.2)	6.1 (2.3–18.8)	2.5 (0.99–7.3)	8.3 (3.5–17.3)	3.4 (1.5–6.6)
20 Gy IFRT (95% CI)	1.06 (0.58–3.0)	0.47 (0.24–1.4)	3.5 (1.3–10.7)	1.4 (0.57–4.1)	4.7 (2.0–9.9)	1.9 (0.86–3.8)

* Excess Relative Risk (ERR) = Relative Risk (RR)-1

† 95% CI = 95% confidence interval

The ERR calculations were performed on three representative patients who had values for the mean female breast dose, mean female lung dose, and mean male lung dose that were closest to the median values of the whole group when treated with 35 Gy mantle field RT.

Thus, for example, moving from 35 Gy mantle RT to 35 Gy IFRT is predicted to reduce the ERR for female breast and lung cancer by approximately 65%, and the ERR for male lung cancer by approximately 35%. Moving from 35 Gy IFRT to 20 Gy IFRT is predicted to reduce ERRs by a further 36% to 43%.

### Doses contributing to the secondary cancer risk

**Figure 4:**
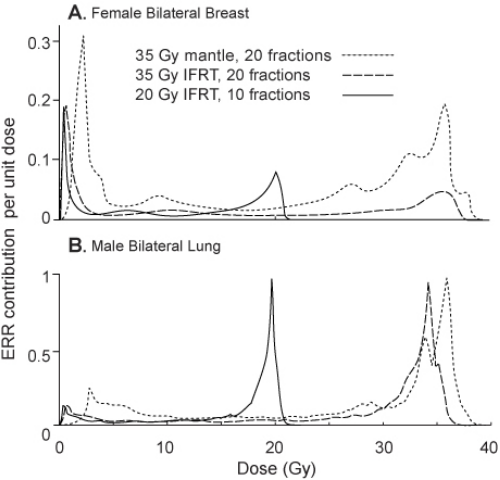
Estimated contribution of different doses within female breast and male lung tissue to the excess relative risk of secondary cancer.

Different parts of each relevant organ are subject to a range of doses, from the prescription dose (or slightly higher) down to low doses. Figure 4 shows the estimated contribution of different doses deposited within a given organ to the estimated ERRs, for two representative cases. The curves are normalized so that the area under each curve is the relevant ERR in Table 3. Both low doses and high doses contributed significantly to the predicted ERR. For the lung, the largest predicted contributions to the total ERR, per unit dose, came from high doses (i.e. close to the prescribed dose), with a small secondary maximum at quite low doses. For the breast, a broader distribution was seen, with the largest predicted contributions per unit dose occurring at total doses of 1–3 Gy, but with significant contributions from a broad range of doses, including a secondary peak at higher doses, near the prescription dose.

## Discussion

Hodgkin lymphoma survivors are known to be at increased risk of radiation-induced SC [1,5,7,29] and cardiovascular disease [2,30,31]. However, published SC risk estimates are primarily derived from HL survivors treated more than 20 years ago with mantle, extended mantle or subtotal nodal RT [1,6,29,32] whereas contemporary RT protocols utilize involved-field (IFRT) given following chemotherapy. To our knowledge, this is the first study to quantify both the reduction in radiation dose to normal tissues delivered with past, current and potential future treatment, and to model the associated reduction in secondary breast and lung cancer risk.

While the motivation for IFRT usage is largely to reduce late effects, in particular SC and cardiac sequelae, quantifying such risk reductions through epidemiological studies is challenging. In particular, the cancer-registry information that proved pivotal in quantifying SC risks after HL [5,7,8] does not generally contain detailed individual-level data on treatment. In contrast, clinical trial datasets contain detailed information regarding initial treatment and may potentially facilitate detailed analyses of the association between specific treatments and SC risk. As noted above, a recent meta-analysis of 10 trials comparing IFRT to EFRT [13] demonstrated no significant difference in SC risk (OR = 1.17 favoring IFRT; p = 0.28), with a similar finding reported in a single BNLI study [14]. A major limitation of clinical trial data, however, is that the specifics of salvage therapy are often not recorded, and the completeness of long-term follow-up and SC reporting may be limited, potentially allowing for misclassification of exposures or outcome. In addition, observational studies cannot predict the potential reduction in SC risk associated with the reduction in IFRT dose to 20 Gy, which may emerge as standard treatment for adult HL [11,12].

We have used a dosimetric risk-modeling approach to second-cancer risk estimation: compared to mantle RT, we have measured the reduction in dose to relevant normal tissues associated with modern IFRT, and then modeled the associated reductions in ERR for radiation-induced breast and lung cancer. The merit of the approach taken here is that it employs both observations from cohort and case-control studies, as well as biological evidence, to predict SC risk based on radiation exposures, without having to wait for decades to observe the actual risk. It is notable that radiation carcinogenicity has historically been modeled primarily as a balance between cellular initiation of malignancy and cellular killing, in which the cancer induction decreases with increasing doses due to greater cell killing [33,34]. In many cases, however, these models predict a reduction in SC risk with RT doses greater than 5–10 Gy, which is clearly contrary to the results of large studies of HL survivors demonstrating increasing risks with escalating doses beyond 20 Gy [5-8,19]. For women diagnosed in their 20's, reported RRs of breast cancer have typically been 3–10 [1,29,35], although higher RRs have been reported in other studies of young women receiving RT [1,6]. Relative risks among women treated in their 30's have been lower, generally consistent with the risk found in this study after 35 Gy mantle RT [1,29]. Similarly, reported RRs of lung cancer typically range from 4–12, with higher RR amongst those treated at younger ages [1,6]. The risks estimated in the 35 Gy mantle scenario in this study are generally in keeping with these published values, providing some external validation of the modeling. In addition, modeled estimates predicted decreasing ERRs of breast and lung cancer with older age at HL treatment, are consistent with the results of several large cohort studies of HL survivors [1,5,7,18,22].

Breast cancer is the most common second malignancy among female HL survivors, particularly those treated at young ages [32]. The reduction in radiation dose and SC risk associated with the transition to IFRT was most evident for the female breast, where the estimated ERR for radiation-induced breast cancer decreased by 64%. This is largely attributable to the smaller volume of breast tissue irradiated when axillary fields are omitted.

Lung cancer remains the most common cause of death from SC following HL [1,8,29]. The transition from mantle to 35 Gy IFRT was associated with a 67% and 36% reduction in estimated ERR of lung cancer in the selected female and male case, respectively. These decreases are largely attributable to the reduction in lung dose with the omission of axillary fields, as well as the more superior placement of the inferior border in IFRT.

The results here suggest that using low-dose IFRT (20 Gy), as opposed to the standard 35 Gy IFRT, would be expected to be associated with further second-cancer risk reduction, with point estimates of the reduction in excess relative risk, in the range from 36–43%. This observation provides a significant support for the rationale behind low-dose IFRT trials currently ongoing [11,12].

Mediastinal RT is also associated with cardiotoxicity [2,31,23]. Hancock *et al *[23] found that HL patients receiving mediastinal RT doses ≥ 30 Gy had a significantly higher risk of cardiac death than those receiving lower doses. For the majority of patients in this study, the transition to IFRT decreased the mean dose to the whole heart significantly but did not reduce the mean dose to the PCA below 30 Gy. This suggests a possible reduction in the incidence of valvular or conduction defects associated with the transition to IFRT. For most patients however, since mean dose to the heart was not decreased, major reductions in the risk of ischemic heart disease will either depend on future dose reductions, or additional volume reductions beyond current IFRT techniques.

This study has limitations that warrant consideration. Firstly, the biologic model applied [19] has inherent limitations, and is based on four assumptions [see Additional file 1]. These assumptions include those regarding estimating risks for m_radiat_, the number of pre-malignant stem cells, dose per fractionation independence, interfraction and post-radiation cellular proliferation. For a more complete explanation see Additional file 1. In addition, there was inter-physician variability in contouring of target volumes and shielding placement for the IFRT plans that may influence the measured dose to normal structures, although its overall effect in this study likely reflects (or underestimates) the heterogeneity that exists in modern clinical practice [36]. In this current study, whole body organ doses were not calculated, and so it was not possible to estimate the reduction in whole body cancer risk. Instead we chose to focus on breast and lung, as these are the two anatomic sites that dominate when considering radiation-induced SC in HL survivors. In addition, ERR estimates were based on only three cases, and although these cases were selected to be representative of the mean dose delivered to breast and lung with 35 Gy mantle RT, the broad distribution of ERR reductions that might be expected in a large population of patients has probably been under-sampled. Finally, we recognize that SC risks involve complex interactions of host, environmental and non-radiation treatment factors. And so while the SC risk estimates presented here consider radiation dose, normal tissue volume, patient age, gender and smoking status, they nevertheless over-simplify these complex interactions [37].

Our results demonstrate that the transition from mantle RT to IFRT and reduced-dose IFRT is associated with significant reductions in radiation dose to normal tissues. Further, modeling results predict that these reductions in radiation exposure will be associated with significant reductions in the risks of breast and lung cancer following IFRT for HL. Ultimately, extended follow-up on patients treated with modern IFRT will be required to definitively quantify the reduction in SC risk associated with this approach.

## Competing interests

The author(s) declare that they have no competing interests.

## Authors' contributions

DCH, ESK, and RT conceived of the study, coordinated the study and helped to draft the manuscript. MP, RKS, DJB, TX, JC participated in data analysis. TTH, MH, NP participated in data collection. All authors read and approved the final manuscript.

## Supplementary Material

Additional file 1 Calculation of Radiation-Induced Cancer Risks from Dose-Volume Histograms using Initiation/Inactivation/Proliferation Methodology. The details provided represent further explanation of the biologic risk modeling applied, including key assumptions, and mathematical estimation of Excess Relative Risk. Click here for file
