# Supplementary material for: A comparison of mantle versus involved-field radiotherapy for Hodgkin's lymphoma: reduction in normal tissue dose and second cancer risk
Source: Radiat Oncol. 2007 Mar 15;2:13. doi: 10.1186/1748-717X-2-13 (PMC1847517; doi:10.1186/1748-717X-2-13)
Supplement: Additional file 1 — Calculation of Radiation-Induced Cancer Risks from Dose-Volume Histograms using Initiation/Inactivation/Proliferation Methodology. The details provided represent further explanation of the biologic risk modeling applied, including key assumptions, and mathematical estimation of Excess Relative Risk. [file 1748-717X-2-13-S1.doc]

Additional File 1

Calculation of Radiation-Induced Cancer Risks from Dose-Volume Histograms

using Initiation / Inactivation / Proliferation Methodology

Summary

It has only fairly recently been clearly established that tissues exposed to fractionated radiation doses as high as 35 Gy are at significant risk for radiation-induced cancer. Early models of radiation-induced cancer had predicted that virtually all radiation-mutated cells would be sterilized by such large doses, and thus the risk of radiation-induced cancer would be minimal. However, recent epidemiological data, showing high risks of radiation-induced cancer at high radiation doses [1-4], have made it apparent that a simple model involving radiation-induction of pre-malignant cells, solely mitigated by cell killing, is not adequate at high radiation doses; consequently, more recent models take into account cellular repopulation by proliferation that occurs both during and after fractionated radiotherapy [5-7]. In terms of carcinogenesis, repopulation largely cancels out the effects of cellular inactivation, the dominant effect being that some of the proliferating cells carry and pass on pre-malignant damage produced earlier in the treatment. Thus, including repopulation in models of radiation-induced cancer results in predictions of substantial cancer risks at high radiation doses, consistent with epidemiological data.

Predictions of dose cancer-risk relationships at high radiation doses were obtained using a recent quantitative mechanistic cell initiation / inactivation / proliferation model [5], which has previously been validated using data on radiation-induced second-cancers in the breast and lung of Hodgkin’s disease patients treated with extended field radiotherapy [1-2,4]. This initiation / inactivation / proliferation model provides a practical methodology for predicting organ-specific cancer risks at high and low radiation doses based on a) cancer risk data from A-bomb survivors (who were exposed to lower doses), b) the demographic variables (age, time since exposure, gender, ethnicity) of the individual(s) of interest, and c) organ-specific parameters describing radiation-induced cellular repopulation (which have previously been estimated both for breast and for lung) [5].

**Assumptions and Detailed Methods**

The initiation / inactivation / proliferation model [5] used here tracks the time development of normal and pre-malignant stem cells during the period from the start of radiation therapy until repopulation is essentially complete. Cellular proliferation begins soon after treatment starts, continues during treatment, and typically continues for a number of weeks after treatment until repopulation is complete.

The main quantity of interest for estimating risks is then *m_radiat_*, the number of pre-malignant stem cells that are a) radiation-initiated or in a lineage originated by a radiation-initiated stem cell, and b) viable at the time repopulation is essentially complete.

*Assumption 1: The excess relative risk (ERR) for radiation induced cancer is proportional to m_radiat_, i.e.,*

*ERR = B m_radiat_ .* (1)

The proportionality factor *B* depends on the time since repopulation is complete (essentially the number of years after radiation therapy), and on other demographic and cohort properties (e.g., age at radiotherapy, sex, and ethnicity). However, *B* does not depend on the dose or dose fractionation of the radiation therapy regimen. Assumption 1 representing the ERR as a product of a dose-dependent term and term depending on cohort properties, is a standard technique (proportional hazards assumption) used in modeling radiation-induced carcinogenesis.

After repopulation has run its full course, there are additional, typically much slower, stages in the carcinogenesis process. The factor *B* in Equation (1) contains the relevant information on these slower processes.

*Assumption 2: Because of its assumed dose / fractionation independence, the factor B in Equation (1) can be estimated from cancer risks derived for atomic-bomb survivors who were exposed to lower doses of radiation than patients treated with radiation therapy.*

To estimate the final quantity in Equation (1), *m_radiat_*, ordinary differential and difference equations are used to track the time development of the mean normal stem-cell number, *n*(*t*), and of the mean initiated, pre-malignant stem-cell number, *m*(*t*), during the fractionated radiotherapy and until cellular repopulation is essentially complete. Suppose that a specific organ receives a spatially-uniform dose *d* in one acute radiation fraction (the actual case where the organ is divided into sub-volumes receiving different doses, as specified by a DVH, will be described below), then

*Assumption 3: The numbers (n^+^ or m^+^) of normal and initiated stem cells just after a radiation fraction are related to the numbers (n- or m-) just before the radiation fraction by:*

*n^+^(k) = S P n-(k), where S = exp (-α d ) and P = exp (-γ d );* (2)

*m^+^(k) = S [m-(k) + (1-P) n-(k)];* (3)

here, *S* is the surviving cell fraction, with the standard parameter α determined from the literature. The parameter γ is the mean number of pre-malignant stem cells produced per unit dose per normal stem cell, so *P* is the fraction of normal stem cells which are not made pre-malignant in one radiation fraction. Equation (3) thus describes the situation where the number of pre-malignant cells just after a dose fraction is the number that survive from just before the fraction, plus the number of cells that are made pre-malignant by, and survive, the dose fraction. In all our calculations, *P* can be approximated as *P=*1 in Equation (2) and as P= 1-γ*d* in Equation (3).

*Assumption 4: Between radiation fractions, and after the last radiation fraction, cellular proliferation is described by the following differential equations:*

*dn/dt = F(n) n, where F(n) = λ [1 - (n/N)] n, λ >0* (4*)*

*dm/dt = r F(n) m, r > 0.* (5)

Equation (4) describes a homeostatic tendency for the mean number of normal stem cells to increase whenever it falls below the original steady-state setpoint number (*N*) of stem cells in the organ. The logistic factor *F(n)* = λ[1-(*n/N*)] ensures that the smaller the number of surviving normal stem cells, the larger the per-cell repopulation rate, and for sufficiently long times the number of normal stem cells approaches its setpoint value *N*.

Equation (5) describes symmetric proliferation of initiated pre-malignant stem cells. The initiation / inactivation / proliferation model assumes that the repopulation dynamics of the pre-malignant stem cells follows the same basic pattern as the repopulation dynamics of the normal stem cells, but with the possibility that the per-cell growth rate of pre-malignant cells differs by a constant factor, *r,* from the per-cell growth rate for normal stem cells.

Equations (2-5) are applied during the period from the start of fractionated therapy until repopulation is essentially complete, some weeks after the last radiation fraction. Using initial values *n=N* and *m=*0, they are solved numerically for the relevant fractionation regimens (for example for 20 fractions, of 1.75 Gy each, daily except on weekends).

The parameter values used in the calculation were the following. For breast, α = 0.18 Gy^-1^, λ= 0.4 day^-1^, and *r* = 0.825. These values for α and λ are the same as those used previously [5]; the value for *r* gives slightly better agreement with the earlier extended-field epidemiological data [4] than the value *r*=0.76 used previously. By a scaling argument, the values of the parameters γ and *N* cancel out of the final results when the methods described below are used. For the lung, we used *r*=1, which gives slightly better fits to the lung data considered previously than does the previously used value *r*=0.96.

To apply Equations (1-5), ERRs are first directly estimated for single radiation exposures at moderate doses, based on cancer incidence data among A-bomb survivors [8]. Second, a well-established methodology described by Land *et al* [9] (and almost identically in the recent BEIR-VII report [10]) is used to adjust dose-dependent ERRs from A-bomb survivors to apply to the demographics of the individuals under study. These two steps are implemented through publicly available on-line software (Interactive Radio-Epidemiological Program, IREP) [11,12]. Finally, the ERR estimates for single moderate-dose radiation exposures are adjusted to fractionated radiotherapy exposures, using the initiation / inactivation / proliferation model outlined here. These two steps allow evaluation of *Bm*_final_ (see Equation (1)) for moderate-dose single exposures. Then the ERR (=*Bm*_final_) is calculated for the fractionated radiotherapy exposure, for the range of doses in the dose-volume histogram, using Equations (1-5).

Finally, given these organ-specific ERR estimates for any given whole-organ dose and fractionation scheme, the DVH data are used to estimate the volume (V) averaged ERRs for radiation-induced breast and lung cancer. Each incremental small volume in the DVH, ΔV_j_ (j=1,200), is associated with a total dose D_j_ = jΔD. Given the associated ERR(D_j_), estimated as described above, the overall predicted ERR is the volume-average of these local ERRs, i.e., ERR = (1/V)Σ _j_ ERR (D_j_) ΔV_j_.

**References**

1. Travis, L.B., Hill, D.A., Dores, G.M., Gospodarowicz, M., van Leeuwen, F.E., Holowaty, E., Glimelius, B., Andersson, M., Wiklund, T., Lynch, C.F., Van't Veer, M.B., Glimelius, I., Storm, H., Pukkala, E., Stovall, M., Curtis, R., Boice, J.D., Jr. and Gilbert, E: **Breast cancer following radiotherapy and chemotherapy among young women with Hodgkin disease.** *JAMA* 2003, **290**: 465-75.

2. van Leeuwen, F.E., Klokman, W.J., Stovall, M., Dahler, E.C., van't Veer, M.B., Noordijk, E.M., Crommelin, M.A., Aleman, B.M., Broeks, A., Gospodarowicz, M., Travis, L.B. and Russell, N.S.: **Roles of radiation dose, chemotherapy, and hormonal factors in breast cancer following Hodgkin's disease.** *J Natl Cancer Inst* 2003, **95**: 971-80.

3. Tinger, A., Wasserman, T.H., Klein, E.E., Miller, E.A., Roberts, T., Piephoff, J.V. and Kucik, N.A.: **The incidence of breast cancer following mantle field radiation therapy as a function of dose and technique.** *Int J Radiat Oncol Biol Phys* 1997, **37**: 865-70.

4. Gilbert, E.S., Stovall, M., Gospodarowicz, M., Van Leeuwen, F.E., Andersson, M., Glimelius, B., Joensuu, T., Lynch, C.F., Curtis, R.E., Holowaty, E., Storm, H., Pukkala, E., van't Veer, M.B., Fraumeni, J.F., Boice, J.D., Jr., Clarke, E.A. and Travis, L.B.: **Lung cancer after treatment for Hodgkin's disease: focus on radiation effects.** *Radiat Res*, 2003, **159**: 161-73.

5. Sachs, R.K. and Brenner, D.J. () **Solid tumor risks after high doses of ionizing radiation.** *Proc Natl Acad Sci USA,* 2005, **102**: 13040-5.

6. Shuryak, I., Sachs, R.K., Hlatky, L., Little, M.P., Hahnfeldt, P. and Brenner, D.J.: **Radiation-induced leukemia at doses relevant to radiation therapy: modeling mechanisms and estimating risks.** *J Natl Cancer Inst* 2006, **98**: 1794-806.

7. Little, M.P. **A multi-compartment cell repopulation model allowing for inter-compartmental migration following radiation exposure, applied to leukaemia.** *J Theor Biol*. 2007, **245** (1):83-97.

8. Thompson, D.E., Mabuchi, K., Ron, E., Soda, M., Tokunaga, M., Ochikubo, S., Sugimoto, S., Ikeda, T., Terasaki, M., Izumi, S. and Preston, D.L.: **Cancer incidence in atomic bomb survivors. Part II: Solid tumors, 1958-1987.** *Radiat Res* 1994, **137**, S17-67.

9. Land, C., Gilbert E, Smith JM, Hoffman FO, Apostoiae I, Thomas B and Kocher D.: **Report of the NCI-CDC Working Group to Revise the 1985 NIH Radioepidemiological Tables.** DHHS Publication 2003 No. 03-5387 NIH Bethesda, MD.

10. NRC. **Health Risks from Exposure to Low Levels of Ionizing Radiation: BEIR VII Phase 2.** 2005 National Academy of Sciences, Washington, D.C.

11. NCI 2003 **Interactive RadioEpidemiological Program, IREP, for estimating assigned share (probability of cancer causation) for exposures to radiation,** Version 5.3. National Cancer Institute, 2006, www.irep.nci.nih.gov.

12. DHHS 42 CFR Parts 81 and 82: **Guidelines for determining the probability of causation and methods for radiation dose reconstruction under the Employees Occupational Illness Compensation Program Act of 2000.** *Federal Register* 2002, **67**, 22296-22314.
